# Large Thyroglossal Duct Cyst Presenting in Adulthood: A Case Report and Literature Review

**DOI:** 10.7759/cureus.37084

**Published:** 2023-04-03

**Authors:** Su Wei Tan, Khairunnisak Misron, Tengku Mohamed Izam Tengku Kamalden

**Affiliations:** 1 Otolaryngology - Head and Neck Surgery, Hospital Sultan Ismail, Johor Bahru, MYS

**Keywords:** thyroid gland, congenital neck mass, midline neck mass, sistrunk operation, thyroglossal duct cyst

## Abstract

Thyroglossal duct cyst (TGDC) is one of the most prevalent congenital midline cervical anomalies which commonly appear during childhood. It has a typically slow-growing presentation with small- or moderate-sized swelling. Here, we described our experience in managing a case of TGDC with an extraordinary clinical course and size, with further evidence from a literature review. A 35-year-old gentleman presented later in adult life with a large midline neck mass of 9.3 x 9.0 x 10.7 cm reaching up to the level of mandible superiorly and sternal notch inferiorly. His history and physical examination, along with fine needle aspiration cytology (FNAC) and CT scan, were suggestive of TGDC. He successfully underwent a Sistrunk operation for TGDC without any morbidity. Postoperatively, he recovered well without evidence of recurrence during follow-up. Large TGDC is always a challenge to clinicians. Correlating the clinical features and imaging is crucial to decide the appropriate surgical treatment.

## Introduction

A thyroglossal duct cyst (TGDC) is one of the most common congenital midline neck masses which accounts for approximately 4.4% of head and neck swelling as evidenced by ultrasonographic studies [1]. It has equal gender preponderance with higher incidence among children below 10 years old [2]. However, there was evidence that the occurrence of TGDC follows bimodal patterns during childhood and adulthood [3]. Embryologically, the development of thyroid glands begins in the fourth week of gestation at the foramen caecum. The thyroglossal duct tracks downward to the posterior surface of the body of hyoid bone and further descends toward its anatomical position to form right and left thyroid lobes which are interconnected by the isthmus. TGDC develops as a result of incomplete involution of the thyroglossal duct which typically disappears in the sixth week of gestation [4].

TGDC usually presents with painless anterior neck swelling near the hyoid bone and exhibits the typical characteristic of moving upward on tongue protrusion during clinical assessment, which could help clinicians differentiate it from a thyroid mass which shows mobility on deglutition. It can occur anywhere from the foramen cecum until the isthmus of the thyroid gland with the infrahyoid being the commonest location followed by the suprahyoid area [1,4]. In one of the local studies, it had been reported that the average size of TGDC was 1.5 cm to 4.0 cm [2]. Although it is a benign lesion, a sudden rapid growth of TGDC is alarming and should raise the suspicion of possible malignancy as it has been reported to have malignant transformation potential in 1% of cases [5]. Here, we present a case of an unusually large TGDC in an adult patient.

## Case presentation

A 35-year-old gentleman with a long-standing history of anterior neck swelling for the past 10 years was prompted to seek medical advice when a previously slow-growing neck swelling had rapidly increased in size within four months following a history of fall whereby the anterior neck hit a blunt surface object. However, he did not sustain any external injuries following the incident. The anterior neck swelling remained painless for 10 years, and he did not experience any dysphagia, respiratory distress, or hoarseness. There was also no history of constitutional symptoms and hypo- or hyperthyroidism. He was subsequently referred to our otorhinolaryngology clinic in a tertiary hospital.

**Figure 1:**
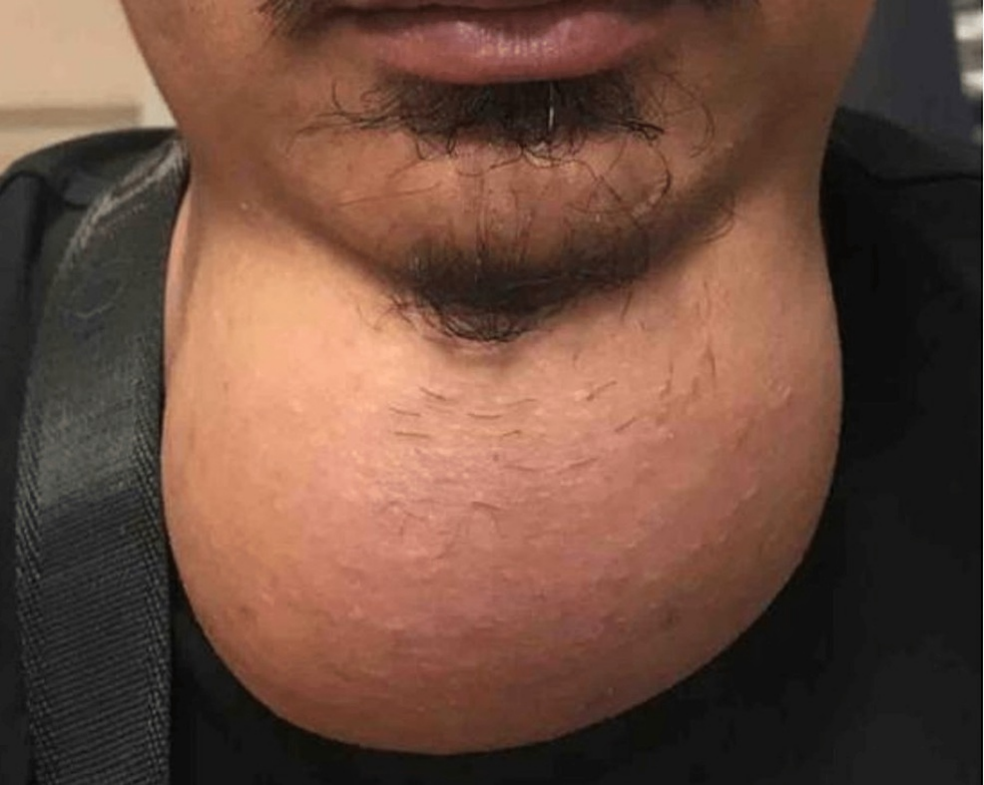
Large anterior neck swelling extending from the mandible superiorly until the level of the sternal notch inferiorly

On examination, he was comfortable with a large anterior neck mass extending superiorly from the mandible until the sternal notch inferiorly (Figure 1).

The neck swelling was soft in consistency, non-tender, and smooth on the surface without discoloration of the skin. The mobility on swallowing or tongue protrusion was difficult to be elicited due to the large neck mass. Laryngoscopy using a 70-degree endoscope (Storz®, Germany) was performed, and there was no sign of an airway compromise or compression of the pharyngeal wall from the mass effect of the anterior neck swelling.

**Figure 2:**
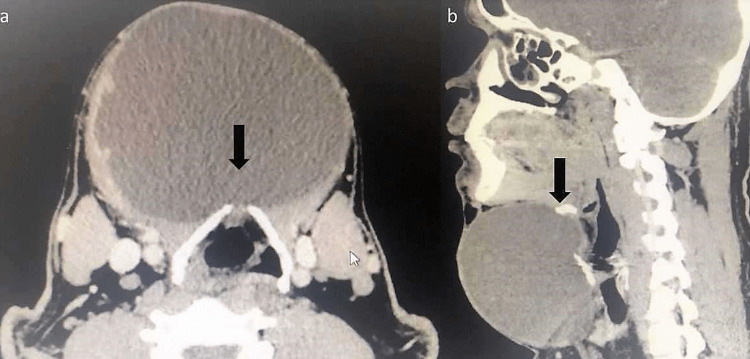
CECT scan of the neck demonstrates a large and well-circumscribed swelling as shown in axial view (a) and sagittal view (b). The swelling attached to the body of the hyoid bone is indicated by the black arrow.

Laboratory investigation for thyroid function test revealed a euthyroid state. Contrast-enhanced computed tomography (CECT) scan of the neck showed a large, well-capsulated cystic mass measuring 9.3 x 9.0 x 10.7 cm with superior attachment at the body of the hyoid bone (Figure 2a, b).

**Figure 3:**
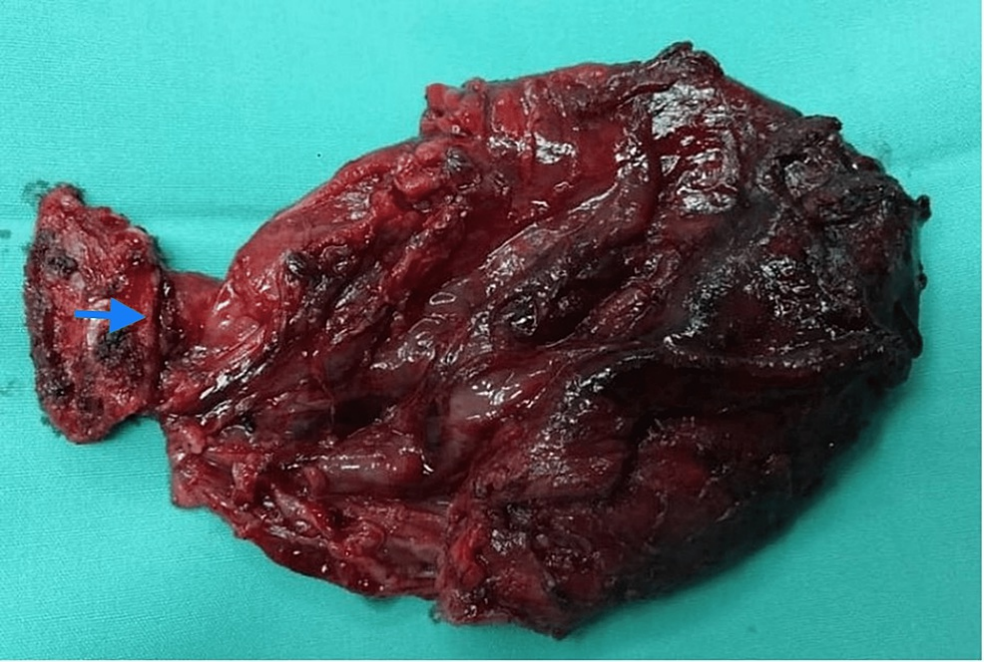
Cyst excised together with the body of hyoid bone as indicated by the arrow

The thyroid gland is present in its usual position. Fine needle aspiration cytology (FNAC) was done and consistent with cystic content without evidence of malignancy. A clinical diagnosis of TGDC was made, and the patient underwent a Sistrunk operation to excise the mass. The mass was completely excised together with the body of the hyoid bone. However, the cyst ruptured during surgical manipulation due to its massive size (Figure 3).

**Figure 4:**
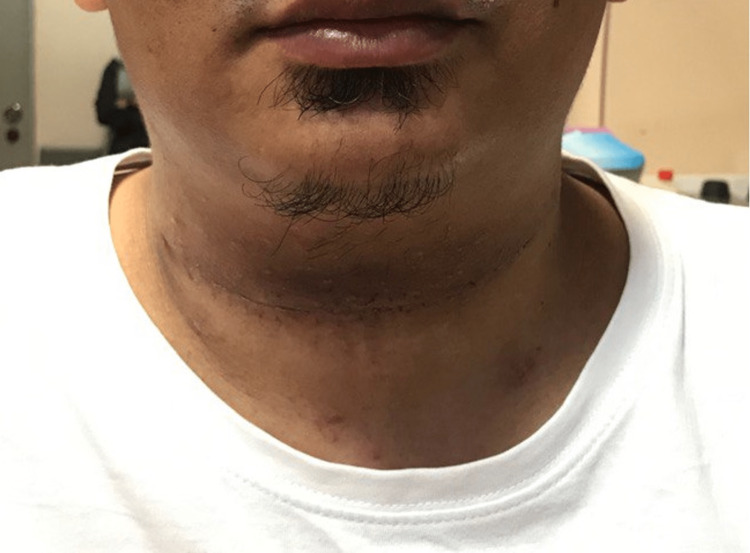
Post-operative image following the Sistrunk procedure

The diagnosis of TGDC was confirmed histologically. He recovered well post-operatively with no further recurrence to date (Figure 4).

## Discussion

**Figure 5:**
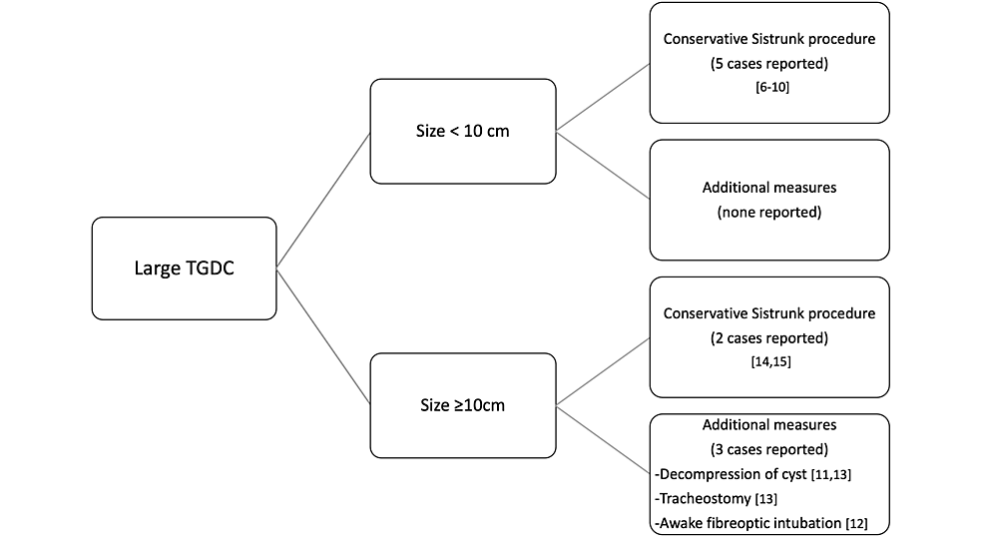
Treatment instituted for large TGDC among adults based on the size in 10 case reports

A TGDC is a benign cystic swelling arising from the embryological remnant of the thyroglossal duct presenting as a midline mass. Although the peak incidence of TGDC is seen in the pediatric age group of 10 years old and below, it can present at any age with a risk of malignant transformation increasing with age [3]. In this case report, our patient presented with an atypical clinical course in terms of the adult age of onset and large cyst size. Several large TGDC cases in adults have been highlighted in the literature [6-15]. Due to its rarity, most of the literature was case reports. Here, we have summarized a few reported cases of large TGDC based on its size and interventions performed (Figure 5).

Although there are various presentations of large TGDCs from the literature review, the common presentation is a slow-growing neck mass rather than compressive symptoms such as in our patient. Given the asymptomatic nature of the mass, some of the patients tend to seek medical attention later until it becomes cosmetically unacceptable or when there are significant obstructive symptoms. Careful preoperative planning is essential in large cysts with compressive symptoms, in anticipation of airway compromise.

Gigantic midline neck mass makes the diagnosis challenging. Although the movement of the mass during protrusion of the tongue is a pathognomonic sign for TGDC, this assessment might not helpful in such a large mass. Few differential diagnoses should be considered such as thyroid mass, lymphadenopathy, extensive dermoid cyst, branchial cyst, or lipoma. The nature of our patient’s presentation with long-standing neck mass which rapidly increased in size over a few months prompted us to consider the possibility of malignancy even though the incidence of malignant transformation was reported to be only 1% [16]. However, the FNAC did not demonstrate any malignant features. The elicited history of a fall in this patient may have been the cause of the sudden rapid growth in a previously slow-growing lesion. Ultrasonography of the neck is sufficient to assess TGDC as well as the presence of a normal thyroid gland. However, in a more complex case or larger cyst such as in our case, a CECT scan is the imaging of choice for proper delineation and extension of the cyst and its adjacent structures as well as to look for calcifications to rule out malignancy [17]. CT scan also plays an important role in assessing the status of the airway, such as narrowing of the airway, due to compression by the cyst. As seen in our case, the CECT findings demonstrated attachment of the mass to the hyoid bone which favors the diagnosis of TGDC, instituting proper treatment accordingly.

The mainstay of treatment for TGDC is surgery which is known as the Sistrunk operation. The principle is based on the embryological development of the thyroid gland. This procedure involves complete excision of the cyst along with the midline body of the hyoid bone where the cyst deeply adhered to this structure to avoid the incidence of recurrence. Righini et al. mentioned that the recurrence rate following the Sistrunk procedure was less than 3% in comparison to simple excision of the cyst [18]. Based on the literature review (Figure 5), reported TGDCs with sizing less than 10 cm were managed solely with Sistrunk operation without necessitating any peri-operative measures [6-10]. In contrast, those sizes 10 cm and larger were more prone to laryngopharyngeal obstruction. Thus, certain peri-operative measures, such as decompression of the cyst or airway intervention, should be considered [11,12]. Iwasa et al. described the technique of aspiration of cyst prior to intubation for large TGDC measuring 18 x 16 cm which resulted in a significant reduction of pharyngeal obstruction and subsequently facilitated the oral intubation [13]. Having said that, Baisakhiya and Abebe et al. described large TGDC > 10 cm without compressive symptoms. Thus, no peri-operative measures were taken [14,15]. Similarly in our case, the pre-operative laryngoscope performed did not reflect any sign of airway compromise or compression of the pharyngeal wall from the mass effect of the anterior neck swelling. Thus, no peri-operative measures were required.

## Conclusions

A TGDC may manifest at any age even though it is more often observed in the pediatric group. Most TGDC has classical presentation, and diagnosis is mainly by clinical judgment. However, large TGDC might pose challenges in diagnosing and treating this condition. Imaging plays an important role in differentiating TGDC from other midline neck masses. Sistrunk surgery has remained the gold-standard management of TGDC. However, we emphasize that careful preoperative planning is essential in large cysts with compressive symptoms, in anticipation of airway compromise.
